# Cultural Resilience and Indigenous Strategies in Dementia Caregiving: Family Caregiver Insights from Uganda

**DOI:** 10.1002/alz70858_096561

**Published:** 2025-12-24

**Authors:** Joy L G Onoria

**Affiliations:** ^1^ Makerere University College of Health Sciences, Uganda, Uganda, Uganda; 7072 mulago hill, Kampala, Kampala, Uganda

## Abstract

**Background:**

Caregiving for individuals with Alzheimer's Disease and Related Dementias (ADRD) in Uganda is shaped by cultural traditions and limited healthcare infrastructure. Family caregivers employ resourceful, culturally informed strategies to address ADRD challenges, often filling gaps left by formal care systems. Understanding these practices is vital for developing inclusive global frameworks that embrace cultural diversity and resilience.

**Method:**

This qualitative study explored caregiving practices among 14 family caregivers in Wakiso District. Participants were purposively sampled to capture diverse caregiving experiences. In‐depth, semi‐structured interviews were conducted in the local language, Luganda, to document caregiving challenges, cultural influences, and symptom management strategies. Interviews were transcribed, translated into English, and analyzed thematically using Braun and Clarke's six‐phase framework. Reliability was enhanced through independent coding by two researchers and member checking with participants.

**Result:**

Caregivers reported a wide range of symptoms, including behavioral challenges (e.g., aggression, wandering), cognitive impairments (e.g., memory loss, hallucinations), mood disturbances (e.g., irritability, depression), and physical difficulties (e.g., feeding refusal, incontinence). A notable observation was the onset of alcohol consumption during wandering episodes among some patients, managed with herbal remedies to induce vomiting. Wandering was often addressed by physically restraining patients to beds or trees for safety.

Agitation and hallucinations were mitigated using cultural and spiritual practices, such as burning calming essences like kakomela, chanting, praying, and using protective amulets (obukomo and hirizi) believed to repel negative forces (empewo). Storytelling, singing, and dancing were used to stabilize mood and foster engagement, while feeding challenges were addressed by involving grandchildren or close family members. Practical solutions included using layered bedsheets as diapers and polythene bags for hygiene. Spiritual interventions, such as communal prayers, blessings, and herbal baths like ebombo, were used to calm patients and strengthen caregiver resilience.

**Conclusion:**

Caregivers in Uganda exhibit resilience and ingenuity in managing ADRD symptoms using culturally rooted practices. This study highlights the potential for integrating these strategies into formal dementia care frameworks to enhance global outcomes. Further research should explore the scalability, effectiveness, and adaptability of these practices to inform equitable, sustainable dementia care policies, particularly in low‐resource settings.

